# Cancer‐testis gene *PIWIL1* promotes cell proliferation, migration, and invasion in lung adenocarcinoma

**DOI:** 10.1002/cam4.1248

**Published:** 2017-11-23

**Authors:** Kaipeng Xie, Kai Zhang, Jing Kong, Cheng Wang, Yayun Gu, Cheng Liang, Tingting Jiang, Na Qin, Jibin Liu, Xuejiang Guo, Ran Huo, Mingxi Liu, Hongxia Ma, Juncheng Dai, Zhibin Hu

**Affiliations:** ^1^ State Key Laboratory of Reproductive Medicine Nanjing Medical University Nanjing 211166 China; ^2^ Department of Epidemiology and Biostatistics School of Public Health Nanjing Medical University Nanjing 211166 China; ^3^ Jiangsu Key Lab of Cancer Biomarkers, Prevention and Treatment Collaborative Innovation Center For Cancer Personalized Medicine Nanjing Medical University Nanjing 211166 China; ^4^ Tumor Biobank Nantong Tumor Hospital Nantong 226000 China

**Keywords:** Cancer‐testis genes, DNA methylation, Epi‐driver genes, Lung adenocarcinoma, *PIWIL1*

## Abstract

Piwi‐like RNA‐mediated gene silencing 1 (*PIWIL1*) has been identified as a novel extremely highly expressed cancer‐testis (CT) gene in lung adenocarcinoma. However, the exact function and mechanism of *PIWIL1* in lung adenocarcinoma remains unclear. Herein, we sought to investigate the role of *PIWIL1* in the occurrence and development of lung adenocarcinoma. We examined the expression pattern of *PIWIL1* in The Cancer Genome Atlas (TCGA) lung adenocarcinoma samples, and validated it by Real‐Time PCR (RT‐PCR) in additional 21 paired lung adenocarcinoma tissues and 16 normal tissues. Subsequently, we explored the biological function of *PIWIL1* in A549 and H1299 cell lines by gain and loss‐of‐function analyses. Using TCGA lung adenocarcinoma data, we further performed coexpression and Gene Ontology (GO) analyses, and analyzed the association of DNA methylation levels in *PIWIL1* promoter region with its expression. Finally, we evaluated its expression in different mutation status of significantly mutated genes (SMGs) in TCGA lung adenocarcinoma data. We observed that *PIWIL1* was expressed in testis and lung adenocarcinoma but not in other normal tissues, and its high expression was associated with shortened survival of lung cancer patients. Overexpression of *PIWIL1* could facilitate the proliferation, invasion and migration of lung adenocarcinoma cells and vice versa. GO analysis revealed that *PIWIL1* upregulated genes were enriched in embryonic development, cell proliferation and regulation of transcription. Moreover, promoter DNA hypomethylation of *PIWIL1* could contribute to its aberrant expression in tumors. Interestingly, *PIWIL1* expression was significantly higher in patients without hepatocyte growth factor (*HGF*) or serine/threonine kinase 11 (*STK11*) mutation (*P *=* *0.006 and 0.005, respectively). *PIWIL1* is an epidriver gene in lung adenocarcinoma, indicating a potential target for further therapy.

## Introduction

Cancer‐testis (CT) genes are a group with limited expression in normal tissues except testis but frequently expressed in various types of cancers, the existence of which indicates the similarities between the processes of gametogenesis and tumorigenesis 1. Owing to the special expression patterns and antigenic properties of CT genes, emerging studies have showed that these genes may be targeted as therapeutic cancer vaccines and biomarkers for early clinical diagnosis and prognosis judgment 2, 3, 4, 5. For example the peptides derived from CT antigens have been used in the clinical trials for several types of cancer, including head and neck cancer and lung cancer 6, 7.To date, more than 200 known CT genes have been identified in the CT database (http://www.cta.lncc.br) 8. Using publically available databases, such as The Cancer Genome Atlas (TCGA), The Encyclopedia of DNA Elements (ENCODE), and The Functional Annotation of The Mammalian Genome (FANTOM), we previously performed a comprehensive analysis to describe the expression characteristics of CT genes and define the extremely highly expressed CT genes (EECTGs) that may be potential epi‐driver genes in 19 cancer types 9. Epi‐driver genes are expressed aberrantly in tumors but not frequently mutated and regulated by DNA methylation or chromatin modification according to the criteria demonstrated by Vogelstein 10. In lung adenocarcinoma, we identified 327 potential EECTGs with testis‐specific proteins expression 9. However, limited studies have explored the function of these EECTGs in cancer.Piwi like RNA‐mediated gene silencing 1 (*PIWIL1*), a lung cancer EECTG in our previous study, is the member of PIWI family and can bind to PIWIL‐interacting RNAs (piRNAs) during spermatogenesis 11. *MIWI*, a murine homolog of *PIWIL1*, encodes a cytoplasmic protein specifically expressed in spermatocytes and spermatids, and controls translation in pachytene spermatocytes and spermatids 12. Accumulating evidence has demonstrated that *PIWIL1* was frequently expressed in various cancers including lung cancer, suggesting the potentially oncogenic roles of *PIWIL1* in the formation or progression of cancer 13, 14, 15, 16. Existing studies mainly focused on the aberrant expression of *PIWIL1* in tumors; however, the biological role of *PIWIL1* in lung cancer has never been elucidated. Thus, in this study, we aimed to examine the expression pattern of *PIWIL1*, and further characterized the biological function and potential regulatory mechanism of *PIWIL1* in lung cancer.

## Materials and Methods

### Patients and specimens

This study was approved by the institutional review board of Nanjing Medical University and informed consent was obtained from all patients included in this study. A total of 21 lung adenocarcinoma patients with pairs of the tumor and the adjacent normal tissues were recruited from the Nantong Cancer Hospital (Nantong City, Jiangsu Province, China), which were histologically or cytologically confirmed by at least two local pathologists. Tissues were frozen in liquid nitrogen after the surgery and stored at −80°C.

### Cell culture

Human lung cancer cell lines (A549 and H1299) were obtained from the Shanghai Institute of Biochemistry and Cell Biology, Chinese Academy of Sciences (Shanghai, China). Cells were both cultured in RPMI‐1640 medium (Gibco, Carlsbad, MA) and supplemented with 100 U/mL penicillin, 100 *μ*g/mL streptomycin and 10% fetal bovine serum (Gibco). These cells were grown at 37°C with 5% CO_2_ in a humidified incubator. A549 and H1299 cell lines were certified by STR genotyping (HKgene, Beijing and MicroRead, Beijing, respectively).

### RNA extraction and Real‐time polymerase chain reaction (PCR) analysis

Total RNA was extracted from the tissues and cells using Trizol reagent (Invitrogen). Approximately 500 ng of RNA was used for the reverse transcription reaction with PrimeScript RT Master Mix (TaKaRa, Dalian, China). Human Multiple Tissue cDNA (MTC) Panels I and II (cat# 636742, 636743; Clontech Laboratories, Palo Alto, CA) were used to validate the *PIWIL1* expression in normal tissues. Using TaqMan Universal PCR Master Mix (Applied Biosystems, Inc.), the cDNA was amplified with probes specific for *PIWIL1* and *ACTB* (Cat. # 4331182, 4331182, Applied Biosystems, Inc., Marsiling, Singapore).

### Protein isolation and western blot

Total cell lysates were prepared with RIPA mixed with 1 mM Phenylmethanesulfonyl fluoride (PMSF). Protein samples (50 *μ*g each) were separated on 10% Tris‐polyacrylagel (Invitrogen) by electrophoresis and blotted onto Polyvinylidene Fluoride (PVDF) membranes. Blots on the membranes were stained with primary antibodies (1:1000, abcam, ab12337 for PIWIL1 and 1:2000, Beyotime, for Tubulin) overnight at 4°C and secondary antibody (Beyotime, Shanghai, China) for 1 h at room temperature. Protein bands were visualized using the ECL Plus western blotting detection reagents (Millipore).

### Overexpression of *PIWIL1* in A549 cells

*PIWIL1* cDNA was cloned into GV358 lentiviral vector (Genechem, Shanghai, China). The 293T cells were transfected with either *PIWIL1* expressed vector or empty vector to produce infectious viruses. Then, the A549 cells were infected with lentivirus, and selected via puromycin (2 *μ*g/mL). The expression of *PIWIL1* in A549 cells was confirmed using western blot.

### SiRNA knockdown of *PIWIL1* in H1299 cells

H1299 cells were transfected with *PIWIL1* siRNA or control siRNA (Ribobio, Guangzhou, China) using Lipofectamine 2000 reagent (Invitrogen, Shanghai, China).The cells were incubated at 37 °C with 5% CO_2_ for 72 h, and collected for further experiments.

### Cell proliferation assay

Cell proliferation was detected by the cell count kit 8 (CCK8, Dojindo, Japan), 5‐ethynyl‐2′‐deoxyuridine (EdU), and colony formation assays. 2 × 10^3^ cells were seeded in 96‐well plates, and 10 *μ*L of reaction solution was added to cells mixed with 100 *μ*L culture medium, the mixture was incubated in 96‐well plates at 37°C for 2 h and then measured for optical density value at 450 nm using a microplate reader (Bio‐Rad, California, USA). According to the manual of EdU labeling/detection kit (Ribobio), transfected cells were cultured in 50 mol/L EdU labeling medium for 2 h at 37°C. Then they were fixed with 4% paraformaldehyde for 30 min and incubated with glycine (2 mg/mL) for 5 min. After washed with PBS, stain these cells with anti‐EdU working solution for 30 min. At last, the cells were incubated with 5 g/mL Hoechst 33342 dye for 30 min. The cell proliferation rate was calculated by the percentage of EdU‐positive cells. In colony formation assay, 200 transfected cells were seeded in 6‐well plates and maintained for 14 days. Colonies were then counted after being fixed with methanol and stained with crystal violet solution.

### Migration and invasion

Cell migration and invasion was investigated by Costar Transwell plates (6.5 mm diameter insert, 8.0 mm pore size, polycarbonate membrane, Corning Sparks, MD). Briefly, transfected cells were plated in the transwells at a density of 2 × 10^4^ cells per 200 *μ*L culture solution and cultured 24 h for migration and 48 h for invasion. Then cells were fixed with methanol and colonies were stained with 0.5% crystal violet for 30 min. The membranes were then dried, inverted, and mounted on microscope slides for analysis. Images of five fields for each membrane (up, down, left, right, and mid) were captured at room temperature via a Q‐fired cooled CCD camera attached to an Olympus microscope and counted by hand with aid of SigmaScanPro imaging analysis software (SigmaScan Chicago, IL). Counts from all fields were averaged to give a mean cell count for each membrane.

### DNA extraction and bisulfite sequencing PCR (BSP)

Genomic DNA was extracted from tissues and cells using the QIAamp DNA Mini Kit (Qiagen, Hilden, Germany). BSP was carried out as previously described 17. DNA (500 ng) was subjected to sodium bisulfite treatment using the EZ DNA Methylation‐Gold^™^ Kit as recommended by the manufacturer (Zymo Research Corporation, CA). Then, the modified DNA was amplified using specific sequencing PCR primers (Forward 5′‐GTTTGGTTTGTTGGGTTTGTG‐3′ and Reverse 5′‐ CCCTAATCCTAATCCTTACACCTC ‐3′). The 410‐bp PCR products, which covered 46 CpG sites (UCSC GRCh37/hg19: chr12:130822264‐130822673) within a CpG‐rich region located in *PIWIL1* promoter and exon 1 were cloned into pMD^®^.19‐T Vector (TaKaRa, Dalian, China). At least six independent colonies of each sample were sequenced by Genscript Corporation (Nanjing, China).

### Bioinformatics analysis

TCGA lung adenocarcinoma RNA‐Seq (level 3, Normalized read counts) dataset of tumor and nontumor tissues was downloaded from TCGA website (https://tcga-data.nci.nih.gov/) on July 15, 2014. The prognostic value of the *PIWIL1* was analyzed by a web‐based meta‐analysis tool – Kaplan–Meier plotter (http://www.kmplot.com/) – which included gene expression and survival data of 2437 lung cancer patients 18. Patients were divided into high expression group and low expression group according to the upper quartile of all samples, and then we calculate the hazard ratio (HR) and 95% confidence interval (CI) using the online tool.To explore the relationship between DNA methylation and *PIWIL1* expression, we downloaded the lung adenocarcinoma methylation data (Infinium HumanMethylation450 Beadchip, level 1) for 448 lung adenocarcinoma tumor samples, and applied the default RnBeads workflow^19^ to reanalyze the DNA methylation levels in the promoter region of *PIWIL1*.Several studies have suggested that significantly mutated genes (SMGs) are considered to be the major source of mutation‐driver genes 20, 21. We further explored the expression levels of *PIWIL1* in different mutation status of 16 lung cancer SMGs which have been reported by the previous study 20. The mutation information of 230 lung adenocarcinoma patients was downloaded from the published study 21.Gene Ontology (GO) function analysis was performed using DAVID Bioinformatics Resources 6.7 (http://david.abcc.ncifcrf.gov/). The RNA sequencing data of 164 samples (87 cancer and 77 corresponding normal tissues) was downloaded from the Gene Expression Omnibus (GEO, https://www.ncbi.nlm.nih.gov/geo/) database. The mRNA and protein expression profiles were downloaded from the Genotype‐Tissue Expression (GTEx, https://www.gtexportal.org/home/) database and the Human Protein Atlas (HPA, https://www.proteinatlas.org/) database.

### Statistical analysis

Normalized read counts of *PIWIL1* in tumor tissues and matched adjacent nontumor tissues were compared using the paired Wilcoxon signed rank test. The Student's *t*‐test was used to assess the differences in treatment groups in functional assays and the *PIWIL1* expression levels in different mutation groups. Spearman's rank correlation coefficient was used to evaluate the correlations between *PIWIL1* expression (log‐transformed) and the promoter DNA methylation or other genes' expression. Significant threshold was set as *P *<* *0.05 in this study.

## Results

### Expression characterizations of *PIWIL1* in lung adenocarcinoma

**Figure 1:**
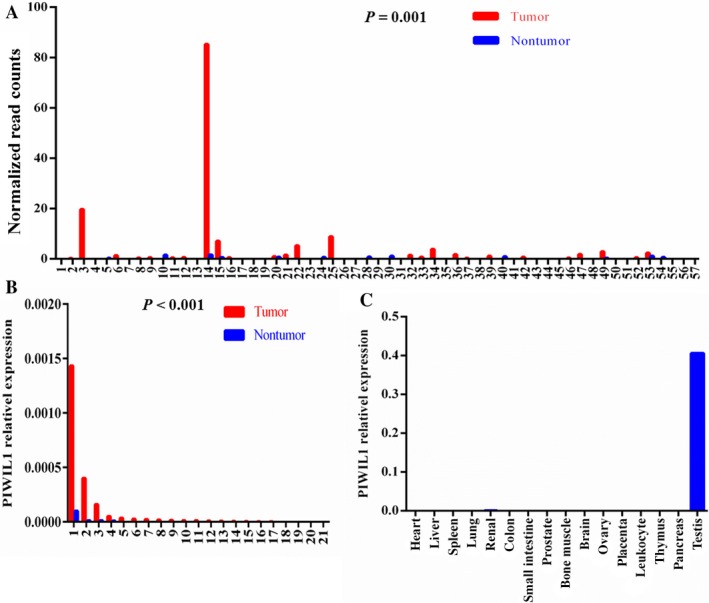
***PIWIL*** *1* was overexpressed in lung adenocarcinoma tissues and the testis tissue. (A) *PIWIL1* mRNA level was significantly higher in lung adenocarcinoma compared with paired normal tissues (*n* = 57) based on the TCGA lung adenocarcinoma dataset. (B) *PIWIL1* expression was significantly increased in tumor tissues compared with matched adjacent normal tissues (*n* = 21) from Nantong Cancer hospital. (C) RT‐PCR verified the testis‐enriched expression of *PIWIL1* from human MTC panels.

We first evaluated the expression levels of *PIWIL1* in TCGA lung adenocarcinoma and observed that *PIWIL1* was overexpressed in tumors compared to normal tissues (*P *=* *0.001, Fig. 1A). Similarly, we observed a striking pattern of *PIWIL1* overexpression in our tumor tissues (*P *<* *0.001, Fig. 1B) by Real‐Time PCR (RT‐PCR). Then, we validated the results in a database (GSE40419) from the GEO database, in which Kim et al. presented the first large scale RNA sequencing study of lung adenocarcinoma and generated RNA sequencing of 164 samples (87 cancer and 77 corresponding normal tissues)^22^. According to this study, we found that *PIWIL1* was overexpressed in lung adenocarcinoma (*P* < 0.001, Fig. S2). Additionally, the results of human MTC panels showed that *PIWIL1* is significantly overexpressed in testis (Fig. 1C). In both mRNA (GTEx) and protein levels (HPA), PIWIL1 was specifically expressed in testis (Fig. S3). Therefore, the specific expression characterizations of *PIWIL1* confirmed that it was a CT gene in lung adenocarcinoma.

Then, we used the Kaplan–Meier plotter tool to assess the relationship between the *PIWIL1* mRNA expression and overall survival (OS) of lung adenocarcinoma patients using TCGA dataset. As shown in Figure S1, high *PIWIL1* expression was significantly associated with a shorter OS in lung adenocarcinoma patients (HR = 1.65, 95% CI = 1.28–2.13; *P *=* *1.0 × 10 ^−4^).

### Overexpression and knockdown of *PIWIL1* in lung adenocarcinoma cell lines

**Figure 2:**
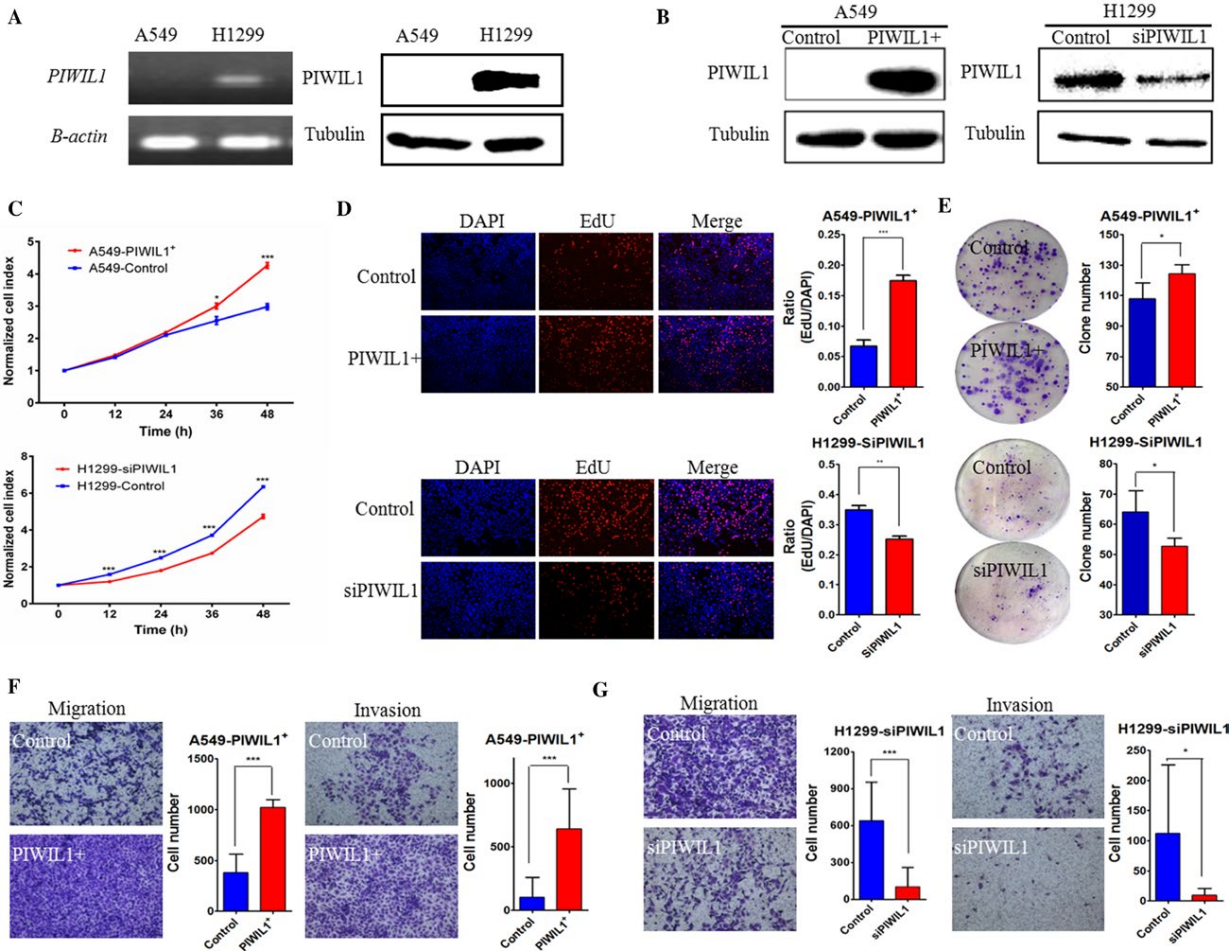
***PIWIL*** *1* facilitated the proliferation, migration and invasion of lung adenocarcinoma cell. (A) RT‐PCR (left panel) and western blot (right panel) demonstrated expression levels of *PIWIL1* in A549 and H1299 cells. *β*‐actin was used as a positive control for RT‐PCR. Tubulin was detected as the internal control for western blot. (B) According to western blot analysis, *PIWIL1* expression in A549 and H1299 cells was significantly increased or inhibited after lentiviral transduction (PIWIL1+) or siRNA transfection (siPIWIL1), respectively. (C) The effects of *PIWIL1* on the cell growth in A549^ex^ ^PIWIL^ ^1^ cells (upper panel) and H1299^si^ ^RNA^ cells (lower panel) measured by CCK8 assay. (D) Cell proliferation was assessed by EdU (Images, 100 × ). The histogram shows the EdU‐positive cells in A549^ex^ ^PIWIL^ ^1^ cells and H1299^si^ ^RNA^ cells (right panel). (E) Colonies were counted in A549^ex^ ^PIWIL^ ^1^ cells and H1299^si^ ^RNA^ cells. (F) Representative images of Migration and (G) Matrigel invasion assays in A549^ex^ ^PIWIL^ ^1^ cells and H1299^si^ ^RNA^ cells (Images, 100×). Quantification of cells was shown at the right. We conducted the experiments in triplicate. Data are presented as means ± SD (SD) (*P <* *0.05, **P <* *0.01, ***P <* *0.001).

To characterize the *PIWIL1* function in lung adenocarcinoma tumorigenesis and metastasis, we examined *PIWIL1* expression in lung adenocarcinoma cells (A549 and H1299) by RT‐PCR and western blot analyses. As shown in Figure 2A, expression of *PIWIL1* was found in H1299 cell line, and loss of expression was found in A549 cell line. Therefore, we overexpressed *PIWIL1* by lentiviral transduction in A549 cells (A549^exPIWIL1^ cells) and reduced *PIWIL1* expression by specific siRNA in H1299 cells (H1299^siRNA^ cells). Efficiency of *PIWIL1* overexpression and knockdown was verified by western blot (Fig. 2B).

Our results showed that the proliferation rate was increased in the A549^exPIWIL1^ cells (Fig. 2C), but inhibited in H1299^siRNA^ cells (Fig. 2C). Similar results were obtained using EdU (red)/Hoechst (blue) immunostaining (Fig. 2D). Furthermore, the results of colony formation assay also revealed that colony numbers of A549^exPIWIL1^ cells were obviously higher than that in those negative control cells (Fig. 2E). Conversely, *PIWIL1* knockdown in H1299 cells attenuated the colony‐forming capability of H1299 cells (Fig. 2C–E). In addition, A549^exPIWIL1^ cells displayed increased cell migration and invasion as compared with controls (Fig. 2F); however, H1299^siRNA^ cells showed decreased cell migration and invasion (Fig. 2G). Taking together, these observations suggested that *PIWIL1* could promote the ability of proliferation, invasion and migration in lung adenocarcinoma.

### Coexpression and GO analyses

To further interpret the effect of *PIWIL1* alteration on lung tumorigenesis, we conducted the coexpression analysis and found a total of 1251 genes significantly correlated with *PIWIL1* expression (false discovery rate)<0.05 and absolute correlation coefficient>0.2). GO function analysis revealed that the positively associated genes were enriched in the embryonic development (*P *=* *1.697 × 10^‐3^), regulation of cell proliferation (*P *=* *6.254 × 10^−3^) and regulation of transcription from RNA polymerase II promoter (*P *=* *8.676 × 10^−3^), while the negatively associated genes were enriched in the regulation of protein modification or metabolic process (all *P *<* *0.05). These findings provided evidence that activation of *PIWIL1* may affect the malignant phenotype of lung adenocarcinoma.

### Identification of *PIWIL1* as an epi‐driver gene in lung adenocarcinoma

**Figure 3:**
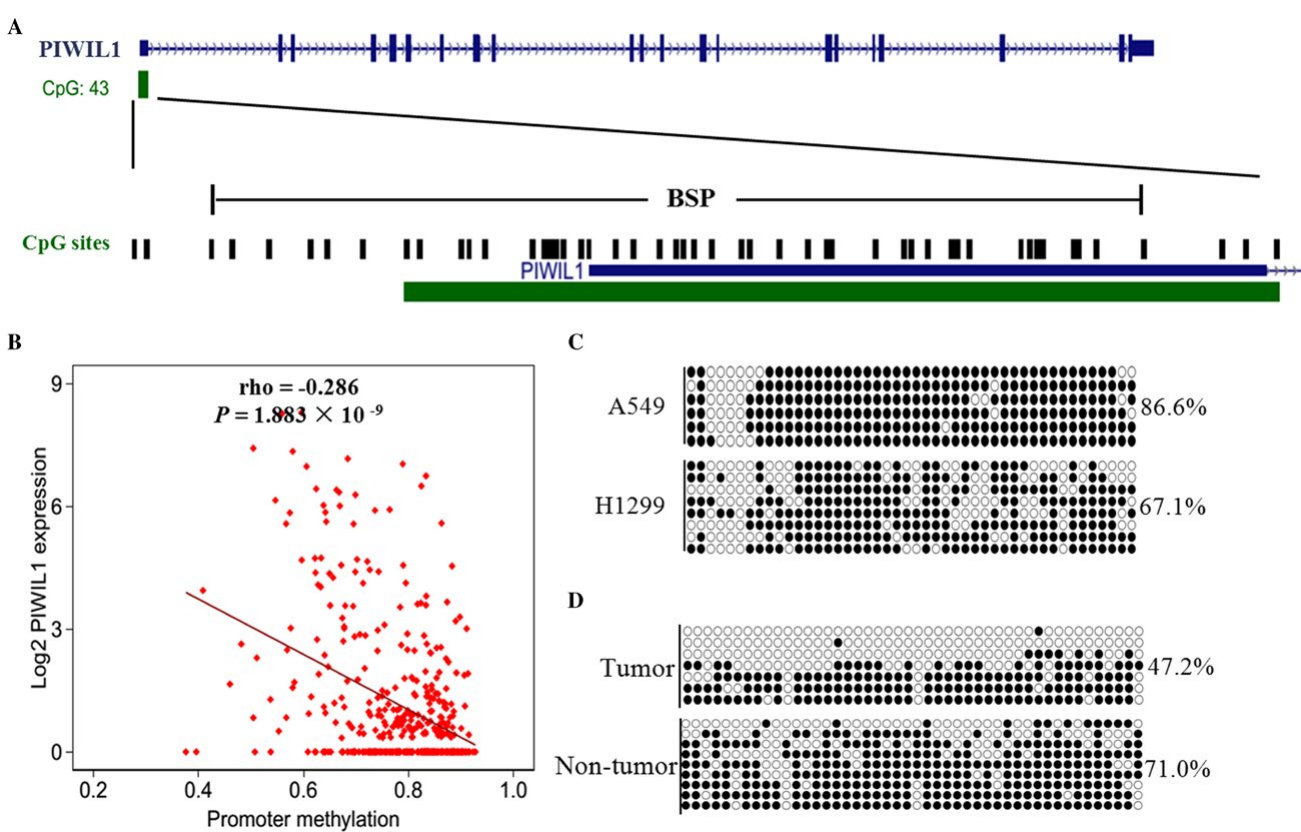
***PIWIL*** *1* expression is regulated by promoter hypomethylation in lung adenocarcinoma. (A) Schematic diagrams showed a CpG island located in the promoter and first exon of *PIWIL1* gene. CpG sites were shown as black bars. The region of CpG sites analyzed by BSP was indicated by a horizontal line marked with BSP, spanning 410 bp. (B) A negative correlation between DNA methylation and *PIWIL1* mRNA level from the TCGA lung adenocarcinoma dataset (n = 426). *ρ*: Spearman's rank correlation coefficient. (C) The methylation status of 46 CpG sites was analyzed by BSP in A549 and H1299 cells and (D) the second paired lung adenocarcinoma tissues. Filled and open circles represented methylated and unmethylated CpG sites, respectively. BSP, bisulfite sequencing PCR.

Since the activation of CT genes in tumors is mediated largely by alterations in DNA hypomethylation 23, we first analyzed the relationship between DNA methylation levels of the 2 kb upstream of *PIWIL1* and its expression levels in 426 lung adenocarcinoma patients based on TCGA database, and found a significantly negative correlation (*ρ* = −0.286, *P *=* *1.883 × 10^−9^, Fig. 3B). Subsequently, we validated the result in lung cancer cells and tissues by BSP (Fig. 3A). We observed the high methylation level in A549 cells (86.6%) with the absent of *PIWIL1* expression and the low methylation level in H1299 cells (67.1%) with high *PIWIL1* expression (Fig. 3C). In one pair of lung adenocarcinoma and adjacent normal tissues (the second paired tissues in Fig. 1B), we confirmed lower DNA methylation levels in the tumor tissue with higher expression levels of *PIWIL1* (Fig. 3D). Thus, these data affirmed that the activation of *PIWIL1* in lung adenocarcinoma may be attributed to the DNA hypomethylation, which suggested that *PIWIL1* might be an epi‐driver of lung adenocarcinoma.

Next, we evaluated the association between *PIWIL1* expression and mutation status of known SMGs of lung adenocarcinoma (Table 1). Our results suggested that *PIWIL1* expression was only significantly higher in patients without hepatocyte growth factor (*HGF*) or serine/threonine kinase 11 (*STK11*) mutation (*P *=* *0.006 and 0.005, respectively).The results further indicated that *PIWIL1* was not activated by mutations in classic mut‐driver genes.

**Table 1:** *PIWIL1* expression levels in different mutation status of SMG

SMG	Mutation	N	Mean ± SD	*P*
*KEAP1*	Yes	32	0.95 ± 1.69	0.772
	No	139	1.04 ± 1.57	
*CDKN2A*	Yes	9	2.63 ± 3.20	0.151
	No	162	0.94 ± 1.41	
*CRIPAK*	Yes	14	1.00 ± 1.94	0.945
	No	157	1.03 ± 1.56	
*EGFR*	Yes	26	1.05 ± 1.86	0.926
	No	145	1.02 ± 1.54	
*EPHA3*	Yes	20	0.93 ± 1.82	0.768
	No	151	1.04 ± 1.56	
*EPHB6*	Yes	23	0.55 ± 0.96	0.127
	No	148	1.10 ± 1.65	
*HGF*	Yes	17	0.53 ± 0.57	**0.006**
	No	154	1.08 ± 1.65	
*KRAS*	Yes	46	1.04 ± 1.96	0.955
	No	125	1.02 ± 1.44	
*MALAT1*	Yes	21	1.08 ± 1.78	0.876
	No	150	1.02 ± 1.57	
*NAV3*	Yes	36	1.06 ± 1.72	0.872
	No	135	1.02 ± 1.56	
*NF1*	Yes	23	1.02 ± 1.12	0.980
	No	148	1.03 ± 1.65	
*SETBP1*	Yes	24	0.93 ± 1.66	0.752
	No	147	1.04 ± 1.58	
*STK11*	Yes	19	0.45 ± 0.74	**0.005**
	No	152	1.10 ± 1.65	
*TLR4*	Yes	25	1.08 ± 1.38	0.825
	No	146	1.02 ± 1.63	
*TP53*	Yes	88	0.87 ± 1.46	0.178
	No	83	1.20 ± 1.07	
*TSHZ3*	Yes	34	1.02 ± 1.54	0.993
	No	137	1.03 ± 1.60	

Bold values represents *P*‐values that are < 0.05.

## Discussion

Our study first provided the evidence for the biological function of CT gene *PIWIL1* in lung adenocarcinoma and demonstrated that activation of *PIWIL1* expression in lung adenocarcinoma cells might facilitate cancer cell proliferation, invasion, and migration. Moreover, the high expression of *PIWIL1* may dependent on its promoter DNA hypomethylation but not the mutations of SMGs.Several studies have revealed that cell fate regulation during spermatogenesis and cell transformation during oncogenesis has shared characteristics, such as immortalization, invasion, induction of meiosis, indicating that aberrant activation of CT genes may drive tumor growth and progression 1, 24. *PIWIL1* is presented in spermatocytes and round spermatids, and plays an important role in spermatogenesis 12, 25, 26. Cox et al. found that *piwi* (*PIWIL1* in Drosophila) is cell‐autonomously required in germline stem cells to promote their division, and overexpression of *piwi* increases the number of germline stem cells 27. Importantly, loss of *PIWIL1* function could lead to the failure of germline stem cell self‐renewal as well as germline cyst formation, egg polarity, and meiosis in Drosophila (*piwi*) model, or the spermatogenic arrest at the round spermatid stage without the development of a sperm tail in null mice (*Miwi*) 12, 28, 29. In this study, we confirmed *PIWIL1* as a CT gene and observed the oncogenic role of *PIWIL1* in lung adenocarcinoma, which were consistent with the findings in other reports 15, 30. For example, Liu et al. reported that overexpression of *PIWIL1* was associated with the proliferation of gastric cancer cells 15. One recent study suggested that overexpression of *PIWIL1* was related to the poor prognosis and stem cell signature in non‐small‐cell lung cancer 16. Moreover, Liang et al. observed that *Hiwi* knockdown could inhibit the growth of lung cancer in nude mice 31. Taken together, these evidences suggested that the activation of *PIWIL1* might be necessary for the tumorigenesis of lung adenocarcinoma.Previous studies have suggested that *PIWIL1* could endow cancer cells with stem‐like properties to acquire a proliferative or metastatic potential during tumor initiation and progression 16, 32. In this study, functional annotation of coexpressed genes based on TCGA lung adenocarcinoma data also indicated that overexpression of *PIWIL1* was associated with embryonic development, cell proliferation, and regulation of transcription. Among these genes, Teratocarcinoma‐derived growth factor 1 (*TDGF1*), also defined as Cripto‐1 (*CR‐1*), is a growth factor with an epidermal growth factor (EGF)‐like domain and plays an indispensable role during embryogenesis and oncogenesis 33. Importantly, numerous studies have demonstrated that *TDGF1* was overexpressed in a wide range of tumors, and contributed to malignant transformation, tumor invasiveness, metastatic spreading, and poor prognosis 34, 35, 36, 37. For instance, two studies have consistently reported that *TDGF1* was significantly higher expressed in lung cancer tissues and related to the poor tumor differentiation, tumor, node, metastases (TNM) stage, and lymph node metastasis 37, 38. Furthermore, serum *TDG*F1 is also a useful diagnosis and prognosis marker for non‐small‐cell lung cancer 39, 40. Additionally, *TDGF1* has been identified as a marker of cancer stem‐like cells 41 and enhances the canonical Wnt/*β*‐catenin signaling pathway by binding to LDL receptor‐related protein 5 (LRP5) and LDL receptor‐related protein 6 (LRP6) coreceptors 35. Therefore, coexpression of *PIWIL1* and *TDGF1* might provide insights into the causal function of *PIWIL1* in lung adenocarcinoma.There is growing evidence showing that activation of CT genes is an early event in tumorigenesis and mediated largely by the change in DNA methylation. For example, MAGE family member A1 (*MAGEA1*), one famous CT gene, has been reported to be activated by the early reduced the promoter methylation in lung carcinogenesis 42. For *PIWIL1*, Ferreira et al. detected the aberrant hypermethylation in the promoter CpG island and diminished expression levels in primary testicular cancer as compared to normal testicular tissues 43. Furthermore, Navarro et al. treated two non‐small‐cell lung carcinoma (NSCLC) cell lines (A549, H23 cells) using the demethylating agent named 5‐aza‐2′‐deoxycytidine (5‐AzadC) and observed a dose‐dependent increase in the *PIWIL1* expression levels 26. Meanwhile, they further found the hypomenthylation in one tumor sample with higher expressed *PIWIL1* and hypermethylation in another tumor sample with lower expressed *PIWIL1* 16. Consistent with these observations, we confirmed that hypomenthylation upregulated the *PIWIL1* expression by BSP in both lung cancer cell lines and tissues in this study.In this study, we also revealed that *PIWIL1* might be an epi‐driver gene in lung adenocarcinoma. Mutations in SMGs could confer selective growth advantage of tumor cells and drive tumorigenesis, which are called mutation driver. However, these mutations cannot completely explain the tumor development and progression 10. Investigators recently proposed that epi‐driver genes compose a major component of the dark matter and may complement alterations in known driver genes 10. In this study, we observed that *PIWIL1* was aberrantly expressed in lung cancer tissues and induced the malignant phenotype. Meanwhile, it is activated by DNA hypomethylation and upregulated in samples without mutations of SMGs. Therefore, we speculated that *PIWIL1* may be an epi‐driver gene in lung adenocarcinoma.In conclusion, we identified that *PIWIL1* was overexpressed in tumor tissues and associated with poor OS in lung adenocarcinoma patients. Further functional experiments and bioinformatics analysis showed that it contributed to the malignant phenotype of lung cancer and was regulated by DNA hypomethylation. Importantly, we identified *PIWIL1* as an epi‐driver gene in lung adenocarcinoma, suggesting its potential target for further therapy.

## Conflict of Interest

None declared.

## Supporting information


**Figure S1**. Kaplan–Meier analysis depicted the association between PIWIL1 expression and the overall survival (OS) of lung adenocarcinoma using Kaplan–Meier plotter based on publicly available data (http://kmplot.com/analysis/)

**Figure S2.** *PIWIL1* mRNA expression level was significantly higher in lung adenocarcinoma compared with paired normal tissues (*n* = 77).

**Figure S3.** *PIWIL1* was specific expressed in the testis tissue in the GTEx and the HPA database (https://www.gtexportal.org/home/ and https://www.proteinatlas.org/, respectively).

**Table S1.** Top 15 enriched biological pathways were positively correlated with *PIWIL1* expression by GO analysis.
**Table S2.** Enriched biological pathways were negatively correlated with *PIWIL1* expression by GO analysis.
