# Supplementary figures and images for: Cancer‐testis gene PIWIL1 promotes cell proliferation, migration, and invasion in lung adenocarcinoma
Source: Cancer Med. 2017 Nov 23;7(1):157–66. doi: 10.1002/cam4.1248 (PMC5774002; doi:10.1002/cam4.1248)

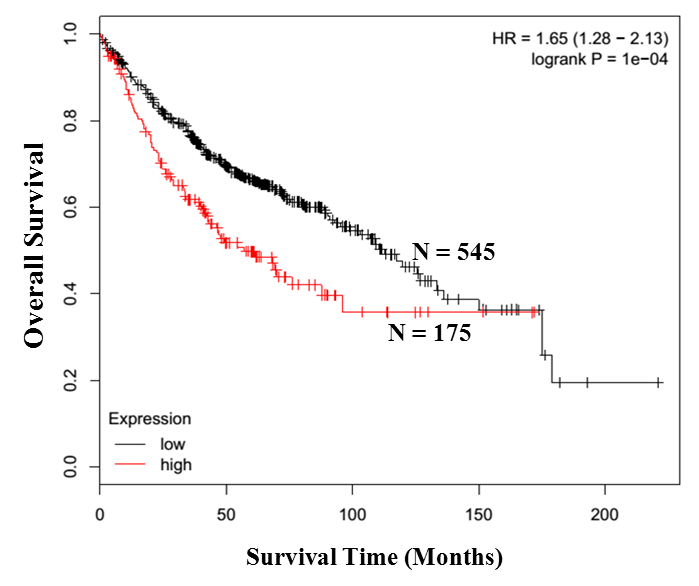

Supplement: Supplementary file 1 — Figure S1. Kaplan–Meier analysis depicted the association between PIWIL1 expression and the overall survival (OS) of lung adenocarcinoma using Kaplan–Meier plotter based on publicly available data (http://kmplot.com/analysis/) [file CAM4-7-157-s001.png]

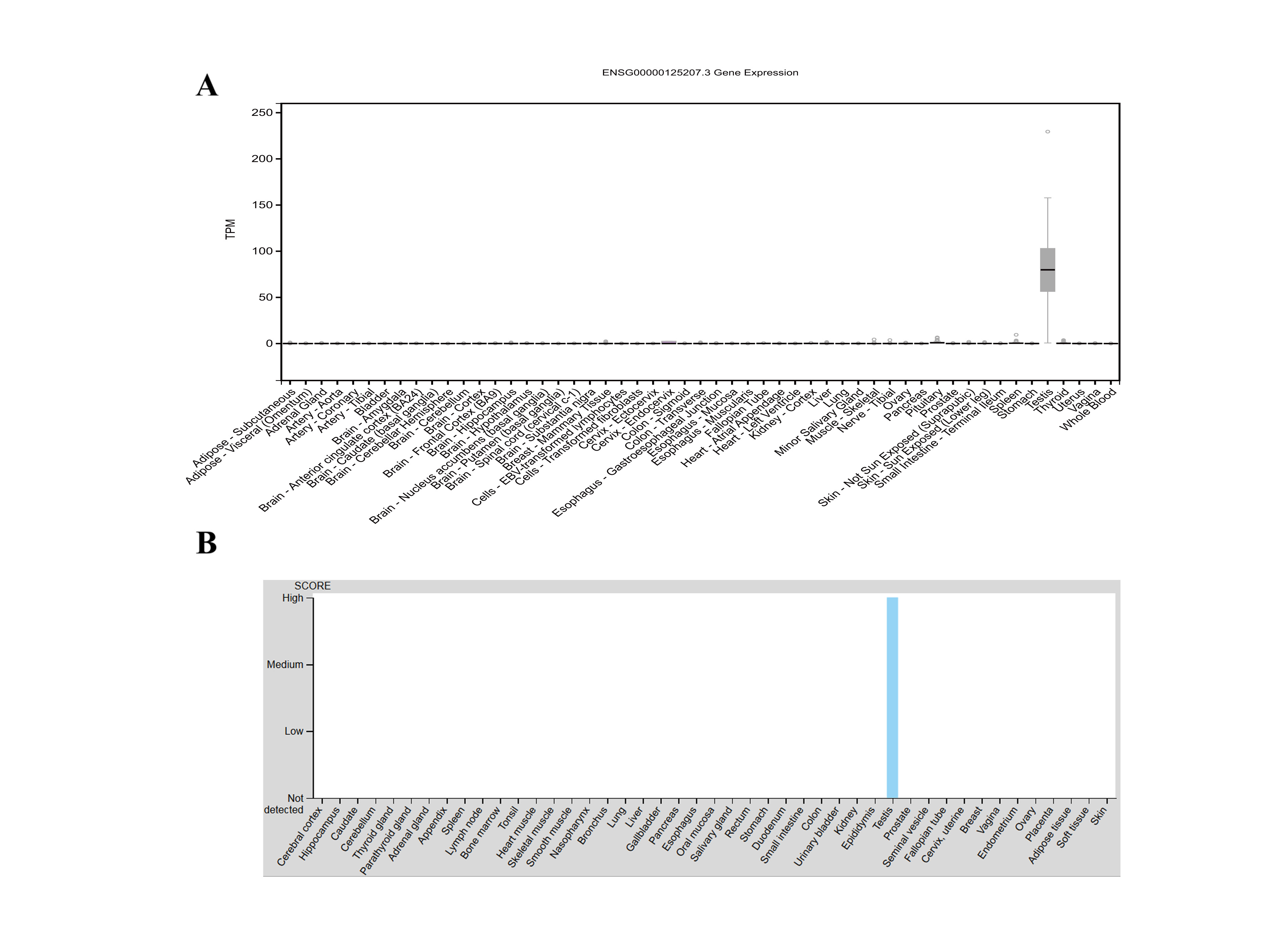

Supplement: Supplementary file 2 — Figure S2. PIWIL1 mRNA expression level was significantly higher in lung adenocarcinoma compared with paired normal tissues (n = 77). [file CAM4-7-157-s002.jpg]

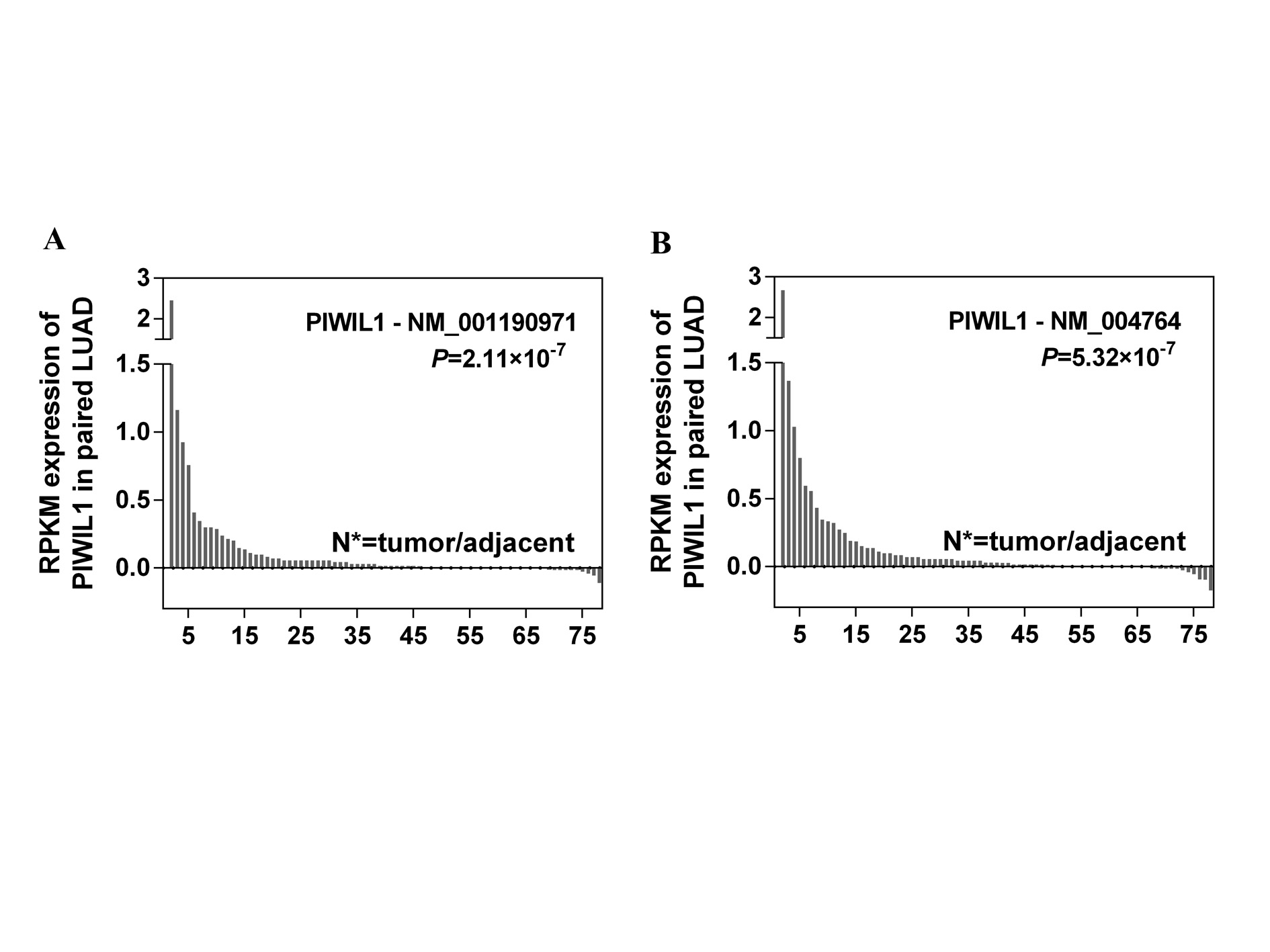

Supplement: Supplementary file 3 — Figure S3. PIWIL1 was specific expressed in the testis tissue in the GTEx and the HPA database (https://www.gtexportal.org/home/ and https://www.proteinatlas.org/, respectively). [file CAM4-7-157-s003.jpg]
