# Supplementary material for: Cancer‐testis gene PIWIL1 promotes cell proliferation, migration, and invasion in lung adenocarcinoma
Source: Cancer Med. 2017 Nov 23;7(1):157–66. doi: 10.1002/cam4.1248 (PMC5774002; doi:10.1002/cam4.1248)
Supplement: Supplementary file 4 — Table S1. Top 15 enriched biological pathways were positively correlated with PIWIL1 expression by GO analysis. Table S2. Enriched biological pathways were negatively correlated with PIWIL1 expression by GO analysis. [file CAM4-7-157-s004.docx]

**Supplementary Tables**

| Supplementary Table 1. Top fifteen enriched biological pathways were positively correlated with *PIWIL1* expression by GO analysis | | | |
| --- | --- | --- | --- |
| **GO Term** | **Description** | ***P* value** | **Genes** |
| GO:0033500 | carbohydrate homeostasis | 1.764E-05 | *SSTR5, HNF1A, FOXA3, PDX1, INSR, PCK1* |
| GO:0042593 | glucose homeostasis | 1.764E-05 | *SSTR5, HNF1A, FOXA3, PDX1, INSR, PCK1* |
| GO:0043009 | chordate embryonic development | 1.603E-03 | *TDGF1,HOXD9, HOXC6, GSC, CDX2, HNF1A, HOXC5, DLL1, PROX1* |
| GO:0009792 | embryonic development ending in birth or egg hatching | 1.697E-03 | *TDGF1,HOXD9, HOXC6, GSC, CDX2, HNF1A, HOXC5, DLL1, PROX1* |
| GO:0003002 | regionalization | 1.996E-03 | *HOXD9, HOXC6, GSC, TDGF1, HOXC5, DLL1, ZIC3* |
| GO:0007389 | pattern specification process | 1.999E-03 | *HOXD9, HOXC6, GSC, CDX2, TDGF1, HOXC5, DLL1, ZIC3* |
| GO:0048878 | chemical homeostasis | 2.092E-03 | *CALCA, SSTR5, CALCB, HNF1A, TRPM8, FOXA3, NPC1L1, PDX1, INSR, ATP7B, PCK1* |
| GO:0009952 | anterior/posterior pattern formation | 2.401E-03 | *HOXD9, HOXC6, TDGF1, HOXC5, DLL1, ZIC3* |
| GO:0030001 | metal ion transport | 3.722E-03 | *TRPM8, SCN3A, ATP2A3, SCN2A, NHEDC2, KCNH8, KCNH3, ABCC8, ATP7B, KCNK10* |
| GO:0042592 | homeostatic process | 3.995E-03 | *HNF1A, TRPM8, FOXA3, PDX1, PCK1, CALCA, CALCB, SSTR5, ID2, NPC1L1, TFF1, INSR, ATP7B* |
| GO:0008284 | positive regulation of cell proliferation | 6.254E-03 | *TDGF1, FGF18, CDX2, ID2, TBC1D8, PDX1, PROX1, INSR, SCG2* |
| GO:0006357 | regulation of transcription from RNA polymerase II promoter | 8.676E-03 | *TDGF1, HOXD9, HOXC6, GSC, CDX2, HNF1A, ID2, HOXC5, NR0B2, PDX1, PROX1, ZIC3* |
| GO:0051094 | positive regulation of developmental process | 1.053E-02 | *CALCA, CDX2, ID2, SERPINF1, SCIN, PROX1, INSR* |
| GO:0006812 | cation transport | 1.116E-02 | *TRPM8, SCN3A, ATP2A3, SCN2A, NHEDC2, KCNH8, KCNH3, ABCC8, ATP7B, KCNK10* |
| GO:0032940 | secretion by cell | 1.230E-02 | *GRM4, HNF1A, NRXN3, SCIN, PDX1, SCG2* |

| Supplementary Table 2. Enriched biological pathways were negatively correlated with *PIWIL1* expression by GO analysis | | | |
| --- | --- | --- | --- |
| **GO Term** | **Description** | ***P* value** | **Genes** |
| GO:0031401 | positive regulation of protein modification process | 1.773E-02 | *ATG10, FBXO4, NHLRC1* |
| GO:0032270 | positive regulation of cellular protein metabolic process | 2.676E-02 | *ATG10, FBXO4, NHLRC1* |
| GO:0051247 | positive regulation of protein metabolic process | 2.893E-02 | *ATG10, FBXO4, NHLRC1* |
| GO:0031399 | regulation of protein modification process | 4.128E-02 | *ATG10, FBXO4, NHLRC1* |

**Supplementary Figure Legends**

Supplementary Figure 1.

Kaplan–Meier analysis depicted the association between PIWIL1 expression and the overall survival (OS) of lung adenocarcinoma using Kaplan–Meier plotter based on publicly available data (http://kmplot.com/analysis/). Patients were divided into high expression group and low expression group according to the upper quartile of all samples.

Supplementary Figure 2.

*PIWIL1* mRNA expression level was significantly higher in lung adenocarcinoma compared with paired normal tissues (n=77). The RPKM expression data was referred to study of Kim et al (PMID: 22975805). The paired Wilcoxon signed rank test was used for the *p* value.

Supplementary Figure 3.

*PIWIL1* was specific expressed in the testis tissue in the GTEx and the HPA database ( https://www.gtexportal.org/home/ and https://www.proteinatlas.org/, respectively).
